# Apigenin ameliorates genitourinary dysfunction in a type 1 diabetic rat model via Drp1 modulation

**DOI:** 10.1038/s41598-024-56395-6

**Published:** 2024-03-08

**Authors:** Mai Khaled, Raghda A. M. Salama, Azza Aboughalia, Mai Tarek, Nesma Mohamed Fawzy

**Affiliations:** 1 Department of Medical Biochemistry and Molecular Biology, Faculty of Medicine, Ain Shams University, Cairo, Egypt; 2 Department of Pharmacology, Faculty of Medicine, Ain Shams University, Cairo, Egypt; 3 Department of Histology, Faculty of Medicine, Ain Shams University, Cairo, Egypt

**Keywords:** Diabetes mellitus, Diabetic nephropathy, Genitourinary complications, Dynamin-related protein 1, Apigenin, Biochemistry, Molecular biology

## Abstract

The present study aimed to explore the potential ameliorative effect of apigenin (APG) against diabetes-associated genitourinary complications in rats. A diabetic rat model was induced by the intraperitoneal injection of streptozotocin (STZ). All experimental animals were treated with vehicle or vehicle plus APG at a dose of 0.78 mg/kg/day for 10 days, either once diabetes was confirmed or at the end of the 3rd week after confirmation of diabetes. Rats were sacrificed at the end of the fifth week. In addition to the histological assessment, an analysis of kidney function tests and serum testosterone was performed to assess diabetic genitourinary complications. Gene expression of the mitochondrial fission protein, dynamin related protein 1 (*Drp1*), was measured in renal and testicular tissues using qRT PCR. APG can increase body weight, reduce blood glucose levels, and improve renal and testicular functions in diabetic rats. APG decreased *Drp1* overexpression in diabetic animals’ kidneys and testes. In summary, our current work discloses that APG attenuates diabetic genitourinary lesions in rats via suppressing *Drp1* overexpression.

## Introduction

Type 1 diabetes mellitus (T1DM) is a chronic metabolic disease that results from an autoimmune attack of the insulin-producing β-cells of the pancreas in genetically susceptible individuals^1^. Diabetic complications represent a major problem in diabetic patients, for example; they are the leading cause of chronic kidney disease (CKD) worldwide^2^. Hyperglycemia promotes the loss of podocytes, endothelial cell hyperfiltration, the expansion of mesangial cells, and thickening of the glomerular basement membrane^3^. Consequently, diabetic nephropathy (DN) is characterized by albuminuria and a reduction in glomerular filtration rate (GFR)^4^.

Moreover, diabetes mellitus (DM) leads to gonadal dysfunction, apoptotic cell death, a decrease in testicular weight, and testosterone production that leads to insufficient production of spermatozoids^5^.

Apigenin (APG; 4′, 5, 7-trihydroxyflavone) is a natural flavonoid widely found in vegetables and fruits^6^. This plant-derived molecule is assumed to have various biological activities such as antioxidant, anti-inflammatory*,* antimutagenic, antiapoptotic, and antitumorigenic in different types of body tissue^7,8^, respectively). Additionally, it has antidiabetic effects as it decreases damage to pancreatic β cells by suppressing oxidative stress and encouraging insulin secretion^9^. The antioxidant property of APG is also responsible for its protective effect on testicular injury in a rat model of ischemia reperfusion injury^10^.

Balanced mitochondrial fusion, fission, and mitophagy are critical to maintaining cell viability. The dynamin-related protein 1 (Drp1) is a cytosolic GTPase protein that mediates mitochondrial fission^11^. Upon activation, it oligomerizes around the outer mitochondrial membrane, initiating its fission and provoking its fragmentation^12^. Assumptions from previous studies suggest that Drp1 promotes Bax/Bak cytochrome c release, which leads to excess fission and ultimately ends with lethal effects on cells up to apoptotic death^13^.

Hyperglycemia provokes certain signaling pathways upstream of Drp1, leading to its overexpression and the loss of normal mitochondrial dynamics^14^**.** Consequently, *Drp1* over-expression has become an obvious finding in DM^15^.

In DM, many signaling pathways regulate *Drp1*, including the P38-MAPK-axis, which is activated by reactive oxygen species (ROS)^11^. Also, PKCδ/Drp1-HK2 and HK2-PINK1/Parkin signal pathways are attributed to mitophagy inhibition by Drp1 upon their activation^11^. On cellular and subcellular levels, podocytes and glomerular mesangial cells display over-expressed *Drp1* during their growth in high glucose media^16^. Thus, exaggerated mitochondrial fission compromises glomerular function and ends with diabetic nephropathy. Moreover, both testes can be affected by *Drp1* overexpression that can be modulated for potential therapeutic purposes^17^.

Based on the aforementioned findings, the present study was carried out to investigate the possible ameliorative effect of APG on genitourinary dysfunction in a type 1 diabetic rat model.

## Material and methods

### Animals

Twenty-eight male Wister rats weighing between 180 and 220 g were obtained from Animal Farm (El-Zyad import office of experimental animals for colleges and research centers, Giza). The rats were housed at the animal house of the Medical Ain Shams Research Institute, Faculty of Medicine at Ain Shams University, and were allowed to acclimatize for one week. They were housed two per cage and subjected to a 12:12-h light/darkness with suitable environmental conditions (temperature 25 ± 2 °C, humidity 55% ± 5%, and good ventilation) and free access to water. The standard rat diet was freshly introduced daily, at 8 a.m. All samples and specimens included in this research were derived from rats (no human participation). Approval for the study was obtained from the ethical committee of the Faculty of Medicine, Ain Shams University, with the approval number.: (FMASU MS 74/2021, FWA 000017585), which operates according to the guidelines of the International Council on Harmonization (ICH) for Medical Science (IOMS), the United States Office for Human Research. The animal experiments and procedures were carried out according to the guidelines of ethical care and standard regulations. We have conducted the experiments in accordance with ARRIVE guidelines^18^. All experimental animals were euthanized by the cervical dislocation method, which is a common method for euthanasia.

#### Induction of type-1 diabetes in rats

Type-1 diabetes was induced by a single intraperitoneal (i.p.) injection of 40 mg/kg b.wt. freshly prepared STZ (STZ; Sigma Aldrich, USA) dissolved in 2 ml of citrate buffer (0.1 N, pH 4.5), following overnight fasting^19^. After 6 h of STZ injection, rats were given a 5% glucose solution to counter the possible hypoglycemic shock (resulting from the sudden release of insulin from the damaged β-cells). The diabetic state was assessed by measuring the fasting blood glucose (FBG) levels from rats’ tails 72 h after STZ injection using a strip operated reflectance meter (Gluco Doctor meter, Korea). The rats with a blood glucose level above 250 mg/dl were considered diabetics^20^.

#### Animal groups

Rats were allocated into four equal groups, as follows: *Group 1:* (Vehicle-injected); rats were subjected to overnight fasting that was followed by a single i.p. injection of 2 ml citrate buffer (used as solvent for STZ). *Group 2:* (Diabetic control); rats in this group were subjected to induction of type-1 diabetes without receiving APG. *Group 3:* (Early APG-injected diabetic group); rats received 0.78 mg/kg/day APG (Sigma Aldrich, USA) dissolved in saline subcutaneously for 10 days^21^, starting once diabetes was confirmed. *Group 4*: (Late APG-injected diabetic group); rats received APG (same dose and duration as group 3) starting at the end of the 3rd week after confirmation of diabetes and the development of diabetic nephropathy as indicated by the deterioration of their kidney function tests.

### Experimental samples

#### Collection of urine samples

The rats were kept in special metabolic cages for the collection of urine samples. Two urine samples were collected throughout the experiment: one by the end of the third week and the other by the end of the fifth week (i.e., before scarification). Each urine sample was kept at 4 °C till assessment of albumin, creatinine, and albumin/creatinine ratio (ACR).

#### Preparation of blood and tissue samples

By the end of the fifth week, rats were weighted and then sacrificed. A midline abdominal incision was made. Blood samples were collected from the abdominal aorta, and then they were left at 37 °C for 30 min. Serum samples were collected after centrifugation and stored at − 80 °C until analysis. Kidneys and testes were dissected and washed with normal saline. Parts of the tissues were kept frozen at − 80 °C until subsequent biochemical analysis, whereas the other parts were fixed in 10% formalin for histopathological evaluation.

### Biochemical analyses

#### Urinary microalbumin and creatinine

Urinary albumin was measured using the rat microalbumin (Malb) ELISA Kit (MyBiosource, USA), whereas urinary and serum creatinine were measured using the QuantiChrom Creatinine ELISA Kit (BioAssay Systems, USA). Urinary ACR was estimated according to the formula: urinary microalbumin (mg/dl)/urinary creatinine (g/dl).

The concentrations of serum urea, FBG and testosterone were measured using (QuantiChrom Urea ELISA Kit (BioAssay Systems, USA), immunoassay Glucose kit (Sigma, USA) and testosterone ELISA kit (CUSABIO, China), respectively, according to the manufacturers’ instructions.

### Molecular analysis

After homogenization of the collected tissue samples by the Cryo Grinder System (OPS Diagnostics; Lebanon, New Jersey), total RNA was extracted from the tissue homogenate using the Spin Vacuum Total RNA Isolation System (Promega, Madison, WI, Catalog code Z3101) according to the manufacturer's instructions. The quantity and quality of total RNA were checked. Equal amounts of total RNA (300 ng) were reverse transcribed into cDNA using the Affinity Script QPCR cDNA Synthesis Kit (Agilent, California; Catalog #600559). The reaction mixtures were incubated in a thermal cycler for 60 min at 37 °C, followed by the inactivation of enzymes at 95 °C for 10 min, and finally cooled at 4 °C. Gene expression levels of *DRP1* were assessed by the SYBR Green-based Real-Time Quantitative PCR method in the ABI Prism 7500 sequence detector system (Applied Biosystems; Foster City, California). Ready-made primers for *Drp1* and *beta-actin* genes were purchased from Qiagen, Germany. All primer sets had a calculated annealing temperature of 60 °C. The reaction mixture was 50 μL consisting of 25 μL of 2 × Brilliant II SYBR Green QPCR Master Mix (Agilent, California; catalog #60083), 2.5 μL primer pair mix (5 PMol/μL each primer), 21.5 μL H_2_O, and 1 μL of cDNA.

The amplification conditions were 2 min at 98 °C for initial denaturation, followed by 40 cycles of 10 s at 98 °C, 10 s at 55 °C, and 30 s at 72 °C. The expression of our target gene was defined based on cycle threshold (Ct) and calculated as 2^−∆∆Ct^ after normalization to the relative expression of the beta actin gene^22^ using Step One Applied Biosystems Software (Foster City).

### Histopathological evaluation

Testicular and renal tissues were fixed in 10% formalin, followed by dehydration, clearing, and embedding in paraffin. Paraffin sections were cut at 4–6 μm thickness and stained by H&E.

For Periodic Acid Achiff (PAS)-stained sections, measurement of PAS% for mesangium was taken by marking the parietal layer of glomerular capsule and excluding the dark color tone for basement membrane of capillaries and red blood cells inside the area and selecting the light color tone only to get the area % of mesangium^23^.

The seminiferous tubules’ diameters were calculated through the averages of the lengths of the short edge and the long edge (two diameters perpendicular to each other), while epithelial thickness was measured by measuring the distances between the sperm closest to the lumen and the basal membrane.

Five specimens from each rat of each group were examined. Five different non-overlapping fields from each specimen were examined. Readings from each field were counted. The sections were examined using magnification 400× for the PAS% of mesangium and epithelial thickness of seminiferous tubules, and using 100× magnification for the mean diameter of the seminiferous tubules.

Measurements were taken by an independent observer blinded to the specimens’ details to get an unbiased assessment. An image analyzer program (Leica Q Win V.3) installed on a computer in the Histology Department, Faculty of Medicine, Ain Shams University, was used for the morphometric measurements. The computer was connected to a Leica DM2500 microscope (Wetzlar, Germany).Photos were taken by Canon EOS 11000 camera at a resolution 1920 × 1280 pixels.

### Statistical analysis

Statistical analyses were carried out and graphically presented using GraphPad Prism version 5.00 for Windows (GraphPad Software, San Diego, California, USA). Data were presented as mean ± SEM. One way analysis of variance (ANOVA) followed by Bonferroni Multiple Comparison tests was performed to compare different groups. Comparisons between groups with repeated time points were carried out using repeated measures 2-way ANOVA. Bonferroni post-tests were used to compare similar time points in different groups. Paired two tailed T test was used to compare the same group in different time points. Results were considered significant at p < 0.05.

### Ethical compliance

All procedures carried out in this study were approved by Ain Shams University Ethical Committee was granted with an autho-rization number: FWA 000017585.

## Results

### Changes in body weight and FBG (from rats’ tails throughout the experiment and from serum after scarification)

In Table 1, the basal body weights of the four groups were matched (P > 0.05). At the end of the 5^th^ week (i.e., just before scarification), only the vehicle-injected rats showed significant increase in their body weight as compared to the starting weight (superscript a indicates p < 0.001).

**Table 1:** Changes in body weight (grams) in the different groups of rats.

Groups	Basal body weight (g)	At 5th week body weight (g)
Vehicle-injected	215.6 ± 11.44	315 ± 6.47^a^
Diabetic control	231 ± 14.42	225.1 ± 17.6
Diabetic + early APG-injection	228.9 ± 4.83	252 ± 24.46
Diabetic + late APG-injection	233.1 ± 10.77	207 ± 13.39

Data are presented as mean ± S.E.M. Superscript a indicates P < 0.001 versus respective baseline value (paired, two tailed t test).

**Figure 1:**
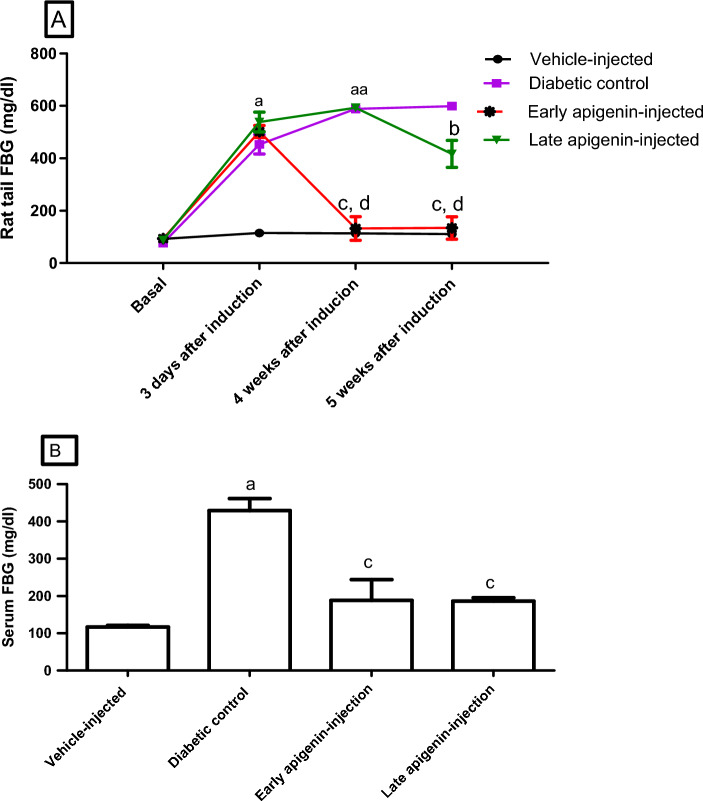
Effects of STZ and APG-injection on FBG from (**A**) rats’ tails, and (**B**) serum after scarification. Data are presented as mean ± SEM. Superscript a indicates p < 0.001 compared to the vehicle-injected group. Superscripts b and c indicate p < 0.01, p < 0.001 respectively compared to the diabetic control group, while d indicates p < 0.001 compared to late APG-injected group (using one way ANOVA, followed by Bonferroni’s multiple comparison post-test).

The comparison of FBG (in blood samples obtained from rats’ tails) of the different groups throughout the whole duration of the study at each time point (Fig. 1A) showed the following: There was no significant difference at baseline; (b) a significant decrease (P < 0.001) in the early APG-injected group (compared to the diabetic control and late APG-injected groups) at the 4th and 5th weeks; (c) significant decrease (P < 0.01) in the late APG-injected group compared to the diabetic control group only at the 5th week.

After scarification, serum FBG level (Fig. 1B) was higher in the diabetic control group compared to the vehicle-injected group (P < 0.001). Also, there was a significant decrease in FBG in the two APG-injected groups compared to the diabetic control group (P < 0.001).

### Effects of STZ and/or apigenin-injection on renal functions and structure, as well as Drp1 mRNA expression

At the 3^rd^ week, there was a significant increase of urinary ACR (mg/g) in the diabetic control than vehicle-injected rats (P < 0.05). At the 5th week, the same ratio was still higher in the diabetic control group than each of the three other groups. Furthermore, each of the early and late APG-injected groups showed reduction of urinary ACR in the 5th than 3rd week (paired two tailed test) (Table 2).

**Table 2:** Urinary albumin/ creatinine ratio (mg/g) in the different groups of rats.

Groups	3rd week	5th week
Vehicle-injected	29.54 ± 2.42	39.72 ± 4.42
Diabetic control	109.3 ± 19.6^a^	141 ± 9.81^b^
Early APG-injection	88.20 ± 12.67	46.88 ± 3.69^c,d^
Late APG-injection	126.5 ± 21.122	58.79 ± 3.03^c,e^

Data are presented as mean ± SEM. Superscripts a and b indicate P < 0.05 and P < 0.001, respectively versus vehicle-injected group, c indicates P < 0.001 versus diabetic control group (One-way ANOVA followed by Bonferroni’s multiple comparison test). d indicates P < 0.01, e indicates P < 0.05 versus respective 3rd week value (paired, two tailed t test).

**Figure 2:**
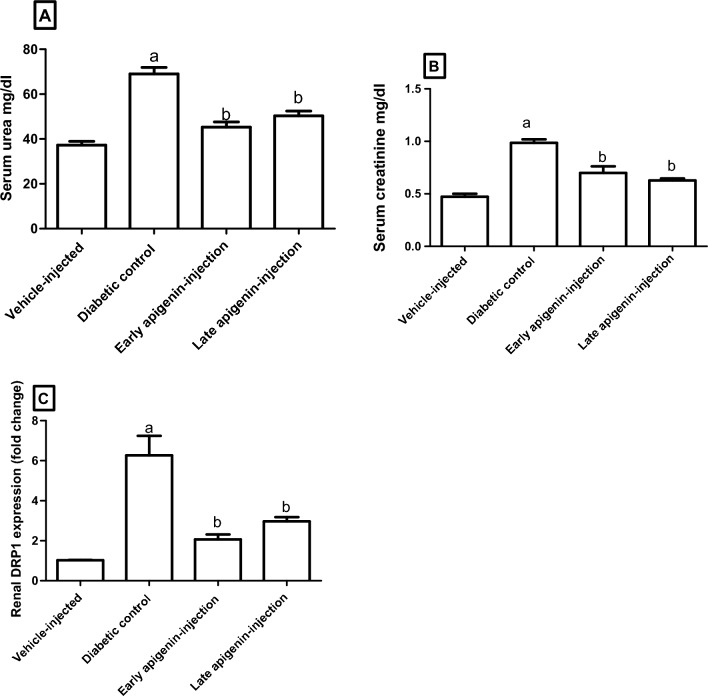
Effects of STZ and APG-injection on (**A**) serum urea, (**B**) serum creatinine, and (**C**) renal *Drp1* expression. Data are presented as mean ± SEM. Superscripts a and b indicate p < 0.001 as compared to the vehicle-injected and diabetic control group respectively (One-way ANOVA followed by Bonferroni’s multiple comparison post-test).

In serum, the concentrations of urea and creatinine were higher in the diabetic control than vehicle-injected rats (Fig. 2A,B). These concentrations were reduced after APG-injection (P < 0.001). However, no statistically significant change was found in relation to the time of APG-injection (i.e., early vs late injection).

The expression of *Drp1* mRNA in renal tissue (Fig. 2C) was higher (six folds) in the diabetic control group. Meanwhile, this elevation of *Drp1* expression was decreased after APG-injection (P < 0.05). Furthermore, the decrease was more evident upon early as compared to late APG-injection, but with lack of statistical significance.

**Figure 3:**
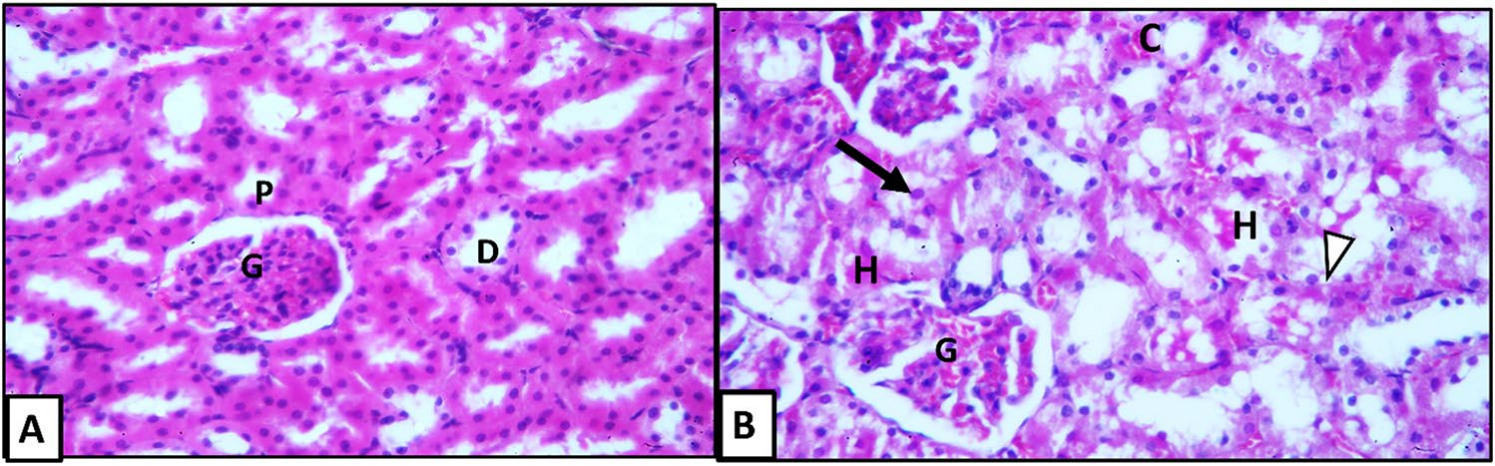
H&E-stained sections of kidney in different groups: (**A**) Vehicle-injected group, (**B**) Diabetic control group (X400). Glomerulus (G); proximal convoluted tubules (P), distal convoluted tubules (D), sloughed tubular epithelium (↑), congested blood vessels (C); Hyalinized acidophilic material (H) and vacuolated tubular epithelium (∆).

**Figure 4:**
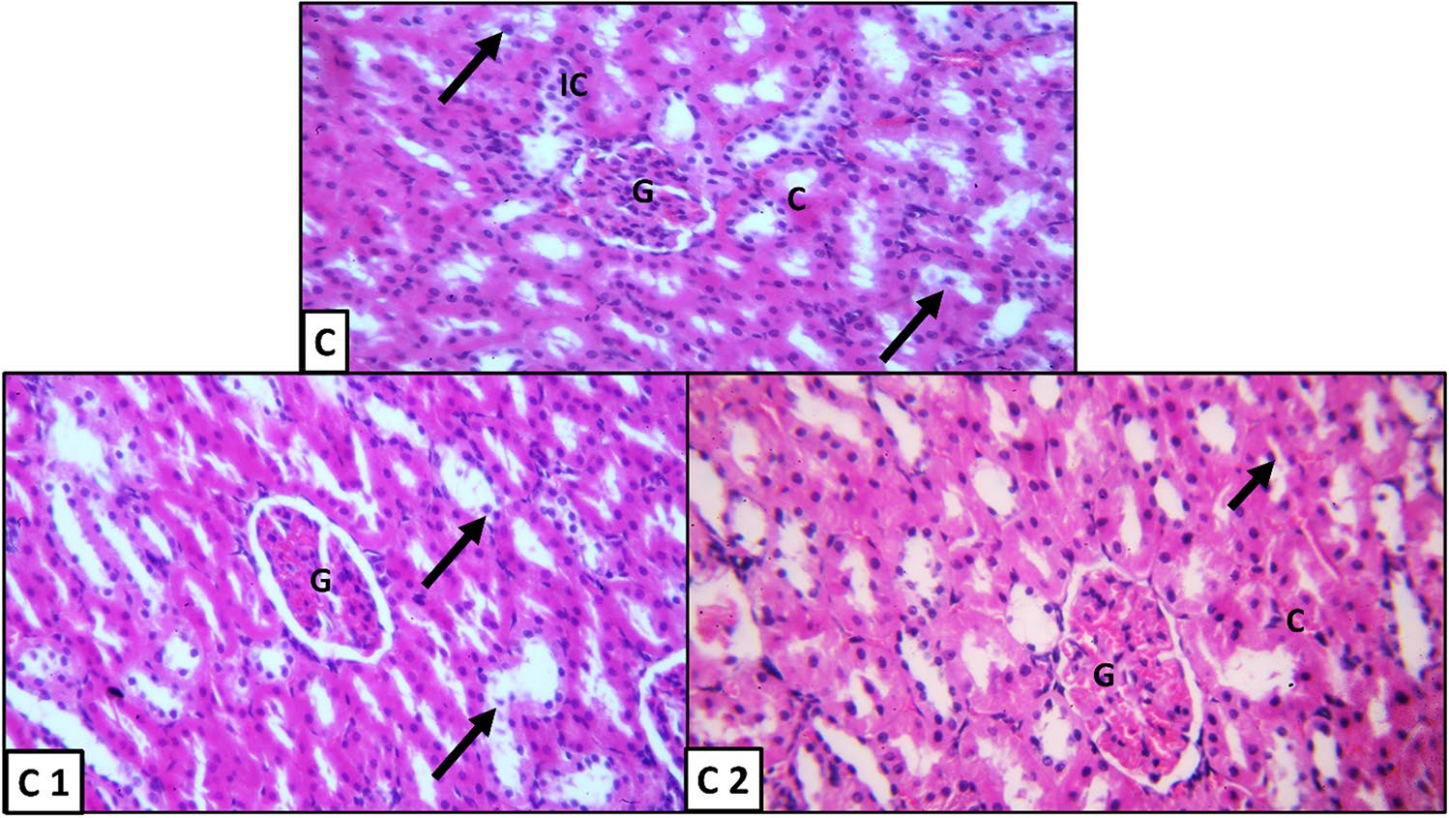
H&E-stained sections of kidney in early APG-injected group: (C, C1, C2) (X400). Glomerulus (G); sloughed tubular epithelium (↑), congested blood vessels (C) and inflammatory cells (IC).

**Figure 5:**
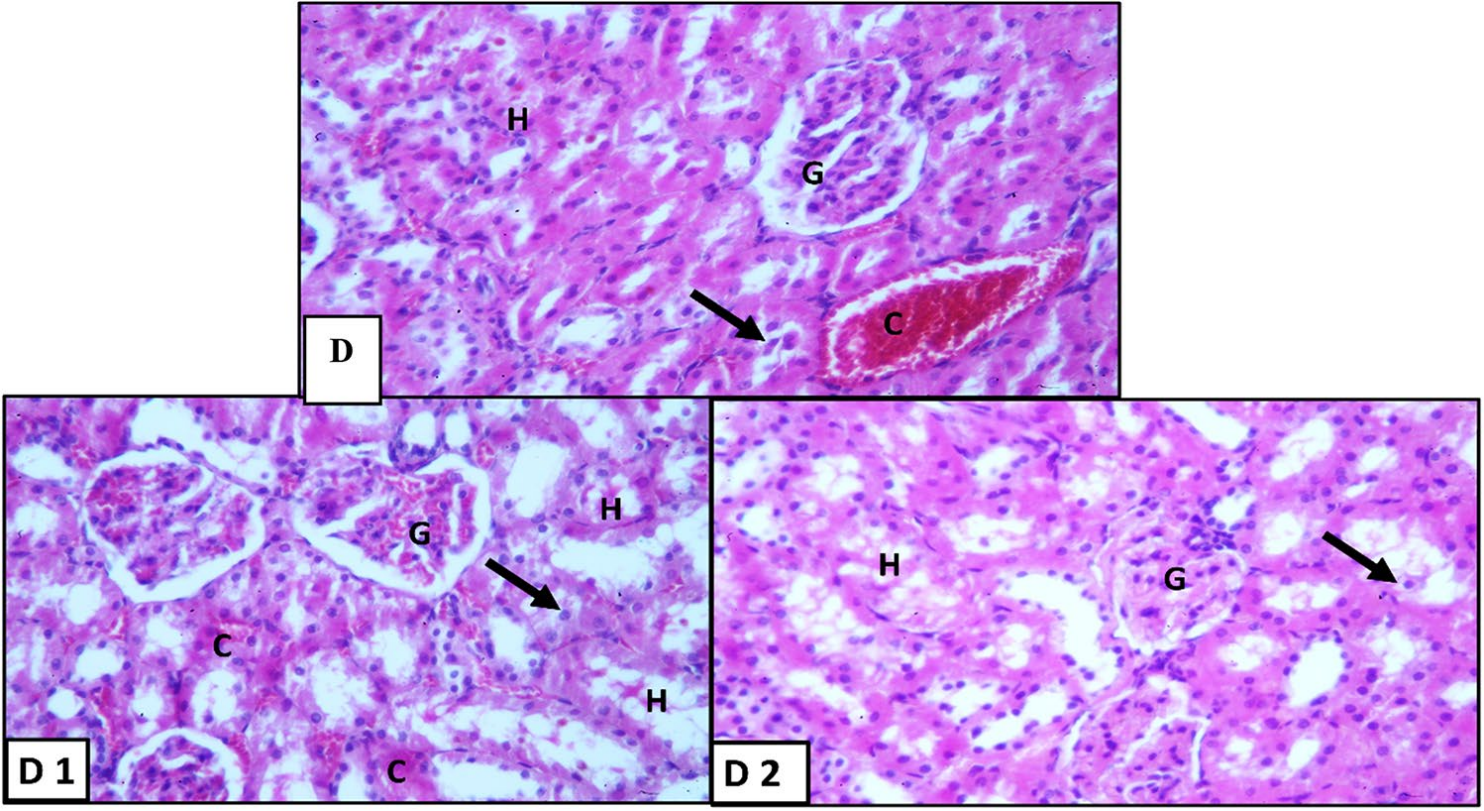
H&E-stained sections of kidney in Late APG-injected group: (D, D1, D2) (X400). Glomerulus (G); sloughed tubular epithelium (↑), congested blood vessels (C); Hyalinized acidophilic material (H) and inflammatory cells (IC).

Examination of H&E-stained sections showed the normal structure of the kidney (Fig. 3A). In diabetic control group (Fig. 3B); renal tubules showed variable degrees of affection. In the early APG-injected rats (Fig. 4), the renal corpuscles and tubules restored the normal structure except for some detached renal tubular epithelial cells, some inflammatory cells and some congested blood vessels. In the late APG-injected group (Fig. 5), some renal tubules were seen with sloughed epithelium. Meanwhile, acidophilic hyaline material in lumen of some renal tubules and congested blood vessels were still seen.

**Figure 6:**
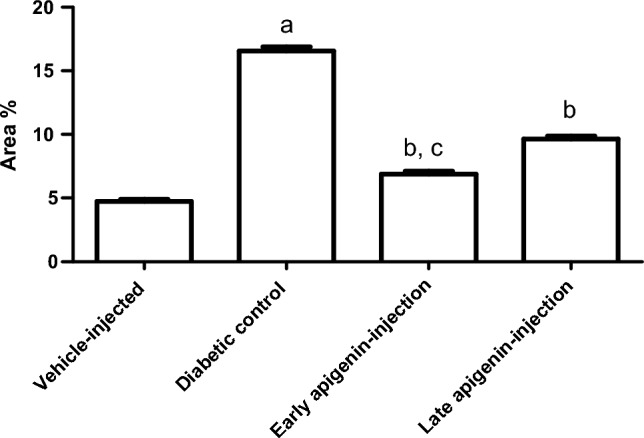
Effects of STZ and APG-injection on PAS area %. Data are presented as mean ± SEM. Superscripts a and b indicate p < 0.001 as compared to the vehicle-injected and diabetic control groups, respectively, while c indicates p < 0.001 upon comparison with late APG-injected group.

**Figure 7:**
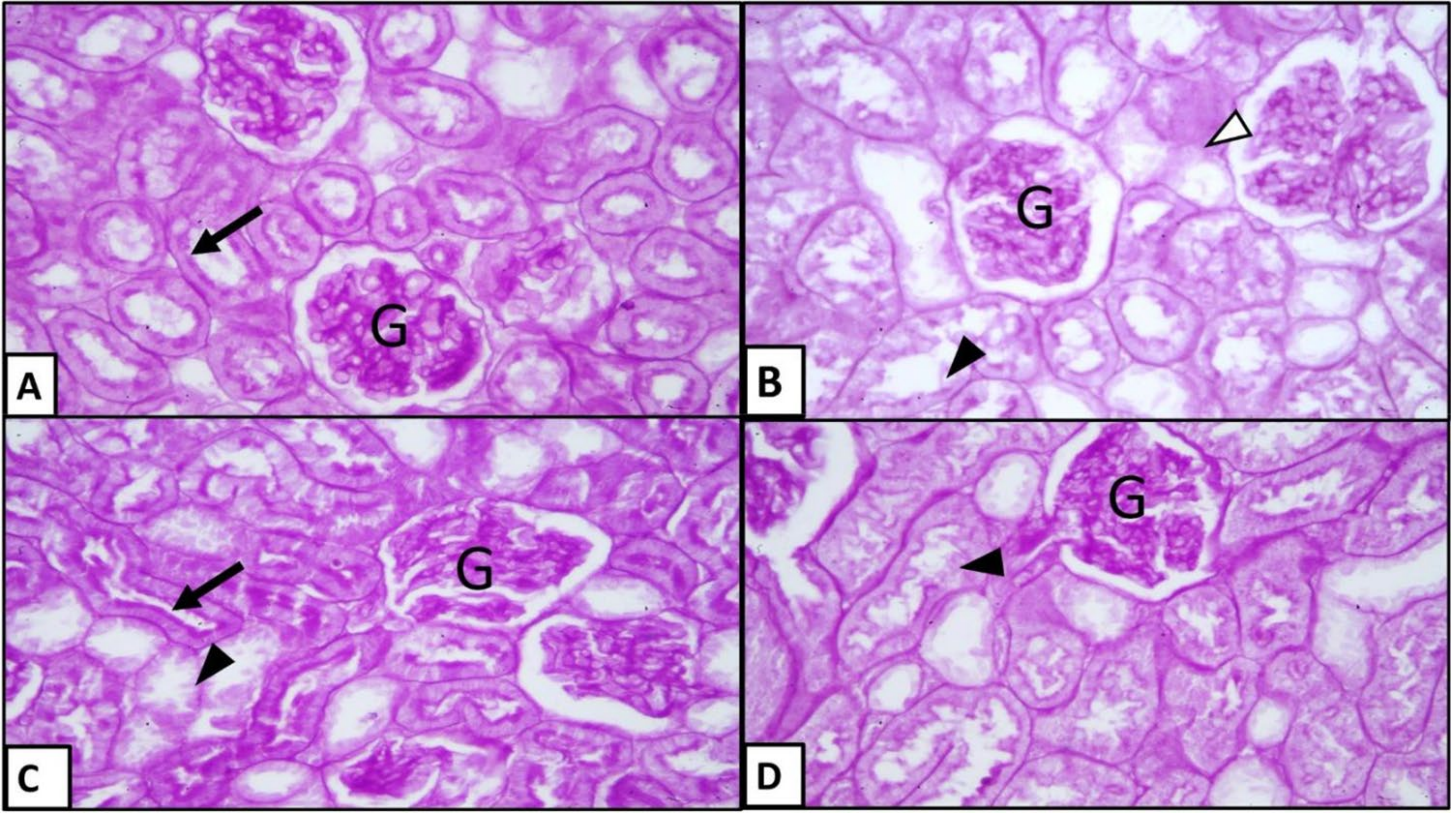
PAS-stained sections of renal tissues in different groups: (**A**) vehicle-injected group, (**B**) diabetic control group, (**C**) early APG-injected group, (**D**) late APG-injected group (X400). PAS positive material in glomerular tuft of capillaries (G); apparent brush border of renal tubules (↑); partial loss of brush border of renal material inside renal tubules (▲); interrupted ill-defined basement membrane of renal tubules (∆).

Staining of renal tissues with Periodic Acid Schiff (PAS) (Fig. 6) showed increase of PAS area % in the diabetic control group (p < 0.001). Renal sections from both APG injected groups revealed % decrease of PAS area in comparison to diabetic control group (Fig. 7). The decrease was more evident upon early APG injection (early vs late) (Fig. 6).

Examination of PAS-stained renal tissue sections from vehicle-injected rats (Fig. 7A); showed PAS positive basement membrane with prominent brush border of proximal convoluted tubules. In the diabetic control group (Fig. 7B); most renal tubules were seen with ill-defined basement membrane and partial loss of brush border. In the early APG-injected group (Fig. 7C); the brush borders of renal tubules were preserved in most of the examined section. However, in the late APG-injected rats (Fig. 7D); the renal tubules were occasionally seen with ill-defined brush borders.

### Effects of STZ and/APG-injection on testicular weight, functions, structure and Drp1 mRNA expression

**Figure 8:**
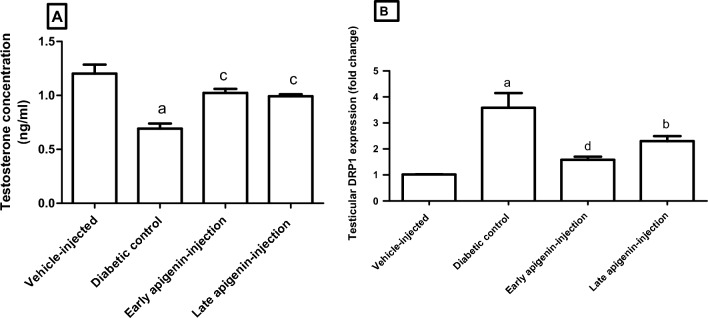
Effects of STZ and/or APG-injection on (**A**) serum testosterone concentration (**B**) and testicular *Drp1* expression. Data are presented as mean ± SEM, where superscript a indicates p < 0.001 compared to vehicle-injected group. Superscripts b, c and d indicate p < 0.05, p < 0.01, and p < 0.001, respectively, upon comparison with diabetic control group.

In spite of the decrease in testicular weight in diabetic control rats compared to other groups, it does not reach a statistical significance (p > 0.05). In (Fig. 8A), the serum concentration of testosterone showed a significant decrease in diabetic control group compared to vehicle-injected group (p < 0.001). Meanwhile, APG-injection (either early or late) elevated serum testosterone compared to diabetic group (p < 0.01). However, there was no significant difference between early and late APG-injected groups.

It was found that testicular *Drp1* expression was significantly up-regulated (about 3.5-folds) in the diabetic control group compared to vehicle-injected group (Fig. 8B). Early and late APG-injection resulted in a significant down-regulation of *Drp1* expression, compared to diabetic control group (p < 0.001, p < 0.05 respectively; Fig. 8B). There was no significant difference between early and late APG-injected groups.

**Figure 9:**
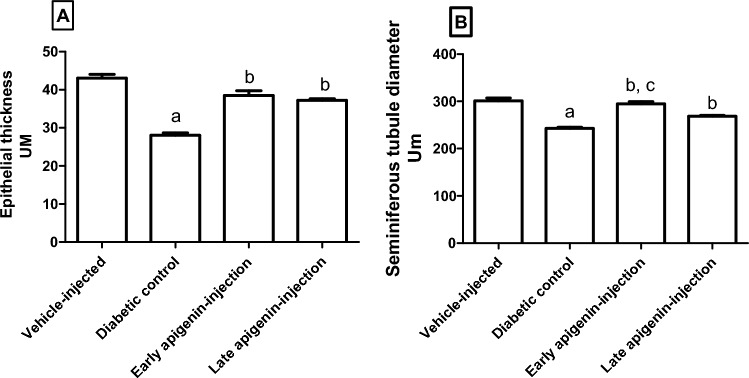
Effects of STZ-induced diabetes and injection of APG on (**A**) epithelial thickness and (**B**) seminiferous tubules’ diameters. Data are presented as mean ± SEM. Superscripts a, b, c indicate p < 0.001 upon comparison with the vehicle-injection, diabetic control and late APG-injection groups, respectively.

There was a significant decrease in the testicular epithelial thickness and seminiferous tubules, diameters in the diabetic control compared to the vehicle-injected rats (Fig. 9A,B). Both parameters (i.e., epithelial thickness and seminiferous tubules, diameters) were improved after APG-injection. Meanwhile, the improvement in seminiferous tubules, diameters was more evident upon early than late APG-injection.

**Figure 10:**
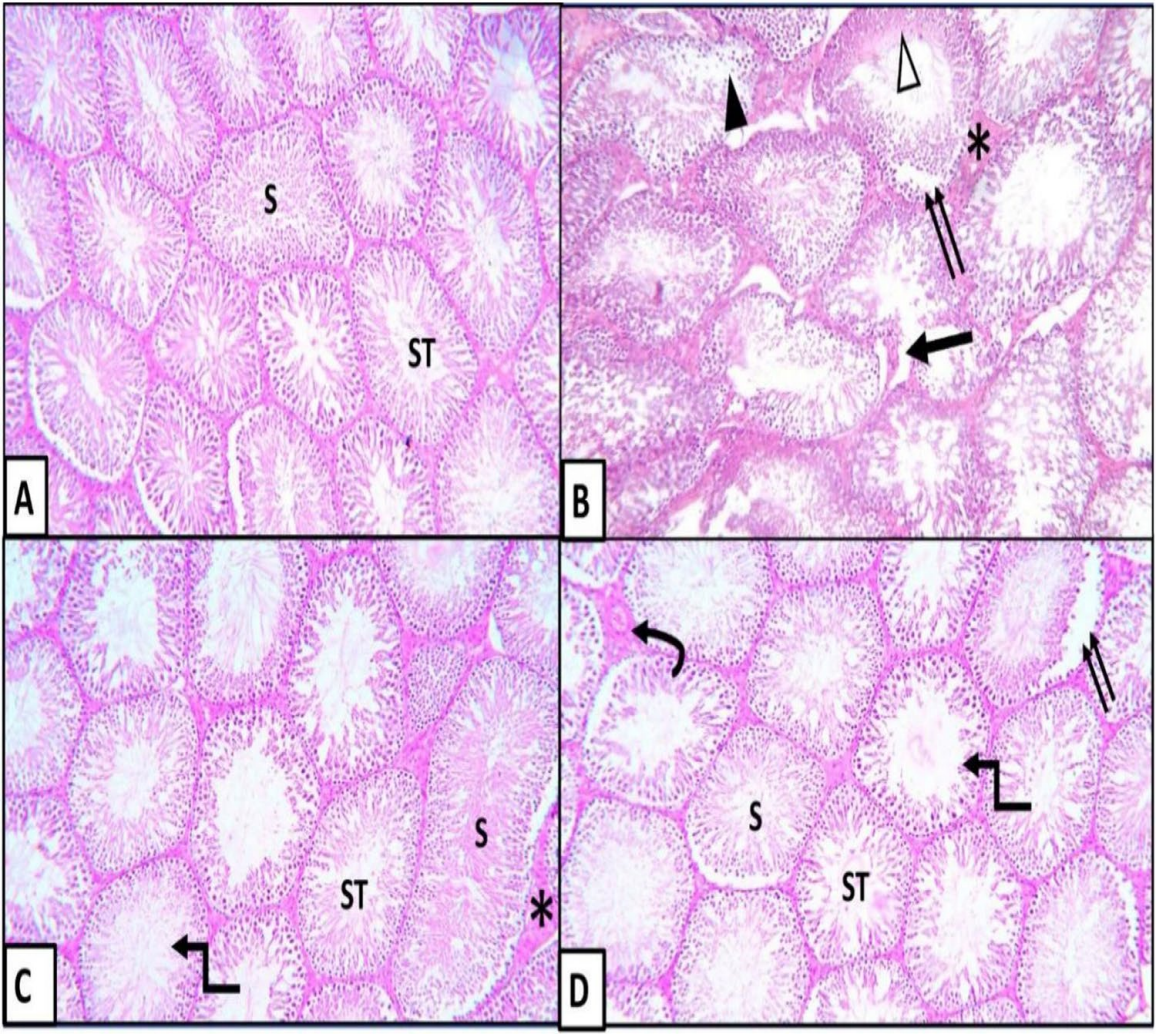
H&E stained sections of testis from different groups of rats: (**A**) Vehicle-injected group, (**B**) Diabetic control group, (**C**) Early APG-injected group, (**D**) Late APG-injected group (X250). N.B: Closely packed seminiferous tubules separated by a narrow interstitial space (ST); spermatozoa inside seminiferous tubule (S); detached spermatogenic cells inside the lumen (▲); discontinuous basement membrane (↑); amorphous acidophilic material inside the lumen (∆); edema in-between the seminiferous tubules (*); separation between spermatogenic cells and basement membrane (↑↑); Congested blood vessels (Curved arrow); seminiferous tubules with wide lumen (Elbow arrow).

Examination of H&E-stained testicular sections from vehicle-injected rats showed normal testicular structure (Fig. 10A) which was distorted in the diabetic control rats, with occasional appearance of sperms within some seminiferous tubules (Fig. 10B). Testicular sections from the early APG-injected rats (Fig. 10C) restored the normal testicular structure except for interstitial edema and wide lumen of some seminiferous tubules. Tissue sections from the late APG-injected rats showed partial restoration of the normal testicular structure. However, some pathological changes can be seen (Fig. 10D).

**Figure 11:**
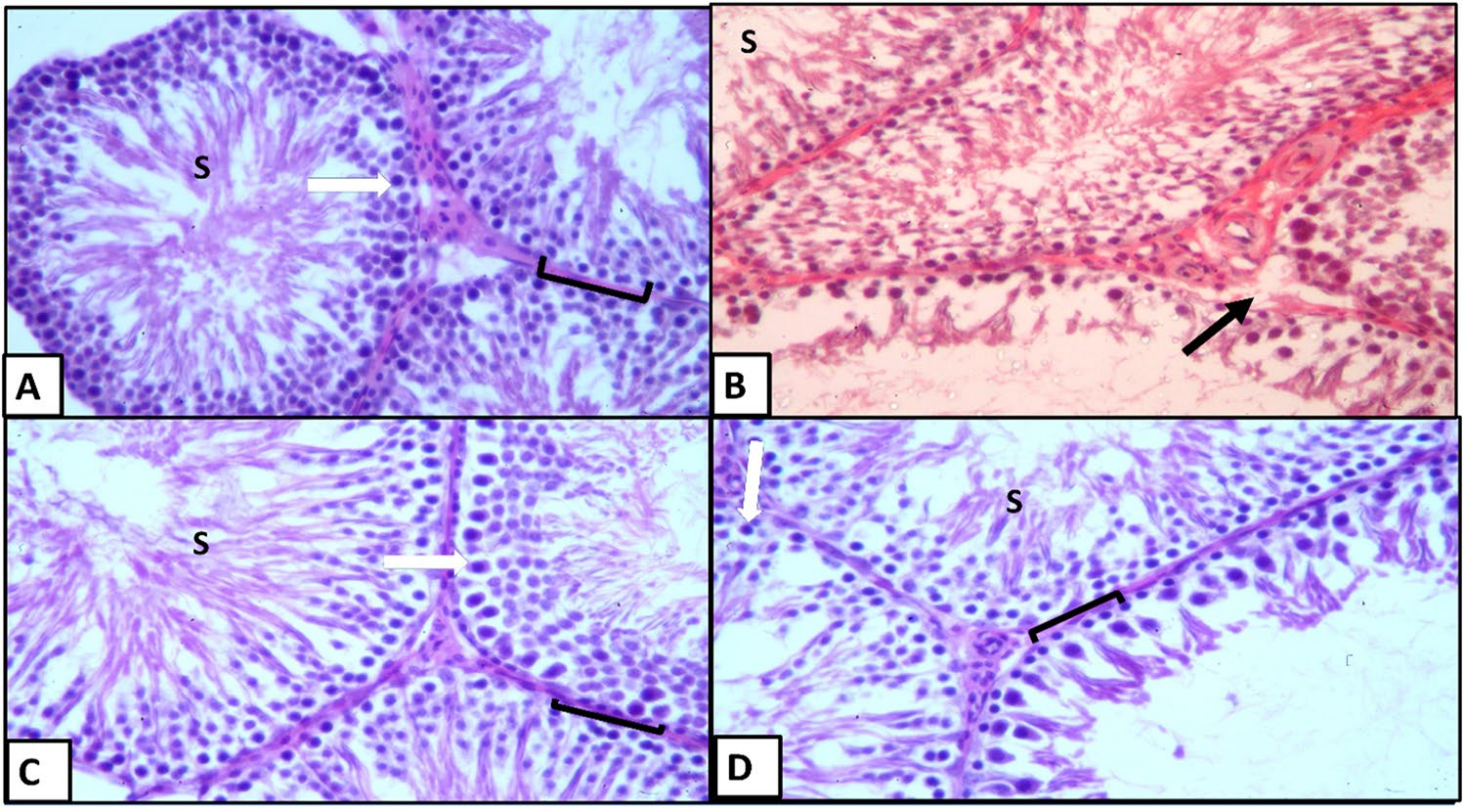
H&E stained sections of testis from different groups of rats at high power: (**A**) vehicle-injected group, (**B**) diabetic control group, (**C**) early APG-injected group, (**D**) late APG-injected group (X400). N.B: Seminiferous tubules lined by spermatogonia ([); primary spermatids (white arrow) and spermatozoa inside seminiferous tubule (S) and discontinuous basement membrane (↑).

At a higher magnification of H&E-stained testicular sections in each seminefrous tubule, the spermatogenic cells included spermatogonia, primary spermatocytes, secondary spermatocytes and early spermatids. Spermatogonia were located in the basal compartment (Fig. 11A,C,D). Primary spermatocytes appeared large in size forming many layers. Secondary spermatocytes were rarely detected. Spermatozoa were detected in the lumen of seminiferous tubules (Fig. 11A,C,D). Meanwhile, this arrangement was highly distorted (Fig. 11B).

The effects of apigenin injection (early and late) in STZ-induced diabetes could be summarized in (Table 3).

**Table 3:** Summary of effects of STZ-induced diabetes and injection of APG in the different groups of rats.

Tissue	Assay	Vehicle-injected	Diabetic control	Early apigenin-injection	Late apigenin-injection
Blood	FBG in serum (mg/dl)	116.9 ± 4.8	429.1 ± 31.76^a^ 267%^#^	188.0 ± 55.8^d^ -56%*	186.3 ± 9.33^d^ -57%* -1%^$^
Kidney	Urinary ACR (mg/g) 5th week	39.72 ± 4.42	141 ± 9.81^a^ 255%^#^	46.88 ± 3.69^d^ -67%*	58.79 ± 3.03^d^ -58%* 25%^$^
	Serum urea (mg/dl)	37.29 ± 1.66	69.00 ± 2.870^a^ 85%^#^	45.29 ± 2.33^d^ -34%*	50.29 ± 2.15^d^ -27%* 11%^$^
	Serum creatinine (mg/dl)	0.47 ± 0.03	0.99 ± 0.03^a^ 111%^#^	0.70 ± 0.06^d^ -29%*	0.63 ± 0.02^d^ -36%* -10%^$^
	Renal Drp1 expression	1.03 ± 0.014	6.27 ± 0.97^a^ 509%^#^	2.073 ± 0.25^d^ -67%*	2.97 ± 0.21^d^ -53%* 43%^$^
	PAS area %	4.74 ± 0.18	16.56 ± 0.34^a^ 249%^#^	6.89 ± 0.23^d^ -58%*	9.64 ± 0.23^d^ -42% 40%^$^
Testis	Serum testosterone (ng/ml)	1.2 ± 0.08	0.69 ± 0.05^a^ -43%^#^	1.02 ± 0.04^c^ 48%*	0.99 ± 0.02^c^ 43%* -3%^$^
	Testicular Drp1 expression	1.02 ± 0.01	3.59 ± 0.56^a^ 252%^#^	1.58 ± 0.12^d^ -56%*	2.30 ± 0.19^b^ -36%* 46%^$^
	Epithelial thickness (μm)	43.06 ± 1	28.06 ± 0.61^a^ -35%^#^	38.51 ± 1.26^d^ 37%*	37.23 ± 0.43^d^ 33%* -3%^$^
	Seminiferous tubule diameter (μm)	301.1 ± 6.13	242.7 ± 2.71^a^ -19%^#^	294.7 ± 4.71^d^ 21%*	268.4 ± 2.15^d^ 11%* -9%^$^

Data are presented as means ± S.E.M, where superscript a indicates p < 0.001 compared to the vehicle- injected group. While superscripts b, c and d indicate p < 0.05, p < 0.01, and p < 0.001, respectively, upon comparison with diabetic control group.

^#^Represents mean % change from vehicle-injected group.

*Represents mean % change from diabetic control group.

^$^Represents mean % change from early apigenin-injected group.

## Discussion

DM is a metabolic disorder with growing global health concerns. Despite the availability of promising treatments, the prevention of tissue damage in the genitourinary system as well as other body systems remains a great challenge^24^. Evidently, STZ is an ideal inducer of diabetes and its complications in rats. It leads to persistent hyperglycemia, which in turn provokes excessive production of ROS with their hazardous effects, especially on mitochondria^25^. The accumulation of evidence indicates that mitochondrial dysfunction contributes to the pathogenesis of diabetic complications^25^.

Impressively, previous studies proved that renal pathological and functional changes in the STZ-induced diabetic rat model are very similar to those seen in the human diabetic kidney, making it an ideal model for research^26^. Consequently, genitourinary complications were provoked in rats by STZ.

Urinary ACR is a reliable biomarker used to screen and diagnose DN, even in its early stages^27^. The results of the present study showed that urinary ACR was elevated (at 3rd and 5th weeks) after diabetes induction by STZ, and serum urea and creatinine were elevated after scarification as well. On a tissue level, H&E stain revealed loss of normal renal architecture, filling of tubules with acidophilic materials, and sloughing of cells. In turn, the increase in PAS area% (mesangial expansion with an ill-defined basement membrane), which is considered another pathognomonic feature of DN^28^**,** was evident in the diabetic group of rats.

Evaluation of genital complications revealed that serum testosterone was decreased in diabetic control rats. Moreover, H&E-stained testicular sections showed that the seminiferous tubules were disorganized, decreased in diameter, and surrounded by edema. Additionally, the number of epithelial cell linings was reduced, and the sections were filled with detached cells. The same findings were also reported by Ma et al.^29^.

APG is a flavonoid with potent antidiabetic benefits due to its ability to suppress α-glucosidase activity, stimulate insulin secretion, and manage of ROS^30^. One of the aims of the current study was to assess the beneficial effect of APG on diabetic genitourinary complications.

Impressively, the results of the current study demonstrated that APG injection was associated with restoration of FBG to its normal value, improvement of urinary and serum kidney function’s tests, and secretion of more testosterone. On the tissue level, APG alleviated the hyperglycemia-induced microstructure damage in the genitourinary system. As the PAS area % was reduced, the diameters of seminiferous tubules were increased, and their epithelia linings were greatly restored.

Previous works revealed the astonishing ability of APG to improve diabetic genitourinary complications from a biochemical and histological perspective^25,31^**.** Concerning the mechanistic pattern, former studies attributed the benefits of APG to a variety of signaling pathways. Most pathways eventually merge on NF-kB (major inflammatory transcription factor), both in the kidney as well as the testis^32–34^). While, with the purpose of DN improvement, Hou et al.^35^ anticipated the miR-423-5P-USF2 axis as a molecular network influenced by APG.

The current work proposes *Drp1* as a novel potential downstream effector of APG in the management of diabetes based on the following: *first*, Drp1 is a mitochondrial fission protein; upon its over-expression and/or over-activation, an imbalance in mitochondrial fission or fusion occurs, which becomes an evident driver in the pathogenesis of diabetes and its genitourinary drawbacks^16,36,37^. *Second*, a plethora of previous works elucidated that Drp1 is not only considered an initiator of diabetes in the pancreas^38^*,* but also a modulator of diabetic complications in body organs such as the brain^39^, eyes^11^, and heart^40^*.* Hence, the current study evaluated *Drp1* expression in the excised kidneys and testicular tissues of the four included groups of rats. Interestingly, our results revealed up-regulation of *Drp1* in both organs excised from the diabetic rat control group. Meanwhile, rats injected with APG displayed lower expression of *Drp1* in these organs. Our results came in accordance with preceding studies approving that *Drp1* is a probable diabetic modulator, hence revealing the impact of mitochondrial dynamics on cell survival.

From a molecular perspective, hyperglycemia and its subsequent elevation of ROS can induce *Drp1* overexpression^41^. Meanwhile, Altara et al.^42^ reported induction of *Drp1* expression by sustained NF-κB activation and vice versa (i.e., blocking NF-κB reduces activity of Drp1 in endothelium). Exploiting the KEGG bioinformatics database (KEGG PATHWAY: map04217) revealed that Drp1 merges with the inflammasome (NLRP3) in the pathway of necroptosis. In the same context, Swanson et al.^43^ and Lu et al.^44^ reported that NLRP3 can be activated by NF-κB with ultimate cell pyroptosis, while it can be blocked by APG in mice, respectively.

Taken together, the current study hypothesized the existence of a novel signaling axis (Drp1/NF-κB/NLRP3) that is involved in diabetic genitourinary complications, and can be suppressed by APG administration.

As regard the timing effects of APG injection, the current study showed improvement in all studied parameters (including Drp1, renal, and testicular functions and structures) upon early as well as late injections. Although the changes were more evident in the early APG-injected diabetic group, they didn’t statistically differ from the late APG-injected one. Thus, we hypothesized that changing the dose, duration, and/or route of APG supplementation may reveal prophylactic as well as therapeutic benefits of APG during the management of diabetic genitourinary complications.

To summarize, the innovative aspect of the current work indicated that APG injection may attenuate diabetic genitourinary complications by means of decreasing blood glucose and improving renal and testicular structures and functions. Thus, the beneficial effects of APG may be mediated by suppressing *Drp1* expression. We further recommend the following: *first,* to investigate the entire members of the pathway hypothesized by this study. *Second,* to evaluate the competing endogenous RNA network that regulates *Drp1* in diabetes with and without APG injection. *Third,* all pharmaceutical aspects (such as dose, duration, route, etc.) of APG should be studied in relation to *Drp1* expression. *Finally,* the proteomics of Drp1 should be assessed in all of the above-mentioned conditions.

## Data Availability

The data that support the findings of this study are available from the corresponding author upon reasonable request.

## Abbreviations

ACR: Albumin/creatinine ratio
APG: Apigenin
Bax/Bak: Bcl-2-associated X protein / Bcl-2 homologous antagonist/killer
CKD: Chronic kidney disease
DM: Diabetes mellitus
DN: Diabetic nephropathy
*Drp1*: Dynamin related protein 1
FBG: Fasting blood glucose
GFR: Glomerular filtration rate
H&E: Hematoxylin and eosin
HK2/PINK1: Hexokinase 2/PTEN-induced kinase1, phosphatase and tensin homologue-induced kinase 1
i.p.: Intraperitoneal
KEGG: Kyoto encyclopedia of genes and genomes
NF-κB: Nuclear factor kappa B
NLRP3: Nod-like receptor protein
P38-MAPK: P38 mitogen-activated protein kinase
PAS: Periodic-acid-Schiff
PKCδ: Protein kinase C delta
qRT PCR: Quantitative reverse transcription polymerase chain reaction
ROS: Reactive oxygen species
STZ: Streptozotocin
T1DM: Type 1 diabetes mellitus
